# Cooperation and group similarity in children and young adults in the UK

**DOI:** 10.1017/ehs.2023.25

**Published:** 2023-09-29

**Authors:** Bonaventura Majolo, Laëtitia Maréchal, Ferenc Igali, Julie Van de Vyver

**Affiliations:** 1 School of Psychology, University of Lincoln, Sarah Swift Building, Brayford Wharf East, Lincoln LN5 7AT, UK; 2 Behavioural Insights and Research team, Magpie, Munro House, Duke St, Leeds, LS9 8AG, UK; 3 Department of Psychology, Durham University, Upper Mountjoy, South road, Durham, DH1 3LE, UK

**Keywords:** culture, development, norms, phenotype, public goods game, social categorisation

## Abstract

For cooperation to be beneficial, cooperators should be able to differentiate individuals who are willing to cooperate from free-riders. In the absence of kin or of familiar individuals, phenotypic similarity (e.g. in terms of language) can be used as a cue of how likely two or more individuals are to behave similarly (whether they will cooperate or free-ride). Thus, phenotypic similarity could affect cooperation. However, it is unclear whether humans respond to any type of phenotypic similarity or whether only salient phenotypic traits guide cooperation. We tested whether within-group, non-salient phenotypic similarity affects cooperation in 280, 3 to 10 year old children and in 76 young adults (mean 19.8 years old) in the UK. We experimentally manipulated the degree of phenotypic similarity in three computer-based experiments. We found no evidence of a preference for, or greater cooperation with, phenotypically similar individuals in children, even though children displayed ingroup preference. Conversely, young adults cooperated more with phenotypically similar than with phenotypically diverse individuals to themselves. Our results suggest that response to non-salient phenotypic similarity varies with age and that young adults may pay more attention to non-salient cues of diversity then children.

## Graphical abstract

**Figure:**
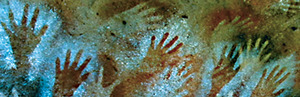

**Social media summary:** Young adults, unlike children, cooperate more in groups composed of phenotypically similar individuals as themselves.

## 1. Introduction

Cooperative behaviours are beneficial when participants share both the benefits (e.g. resource acquisition) and costs (e.g. time spent coordinating the behaviour of all participants) of cooperation (Bowles & Gintis, 2011; Noë, 2010). However, when cooperation produces goods that cannot be monopolised by cooperators (e.g. taxpayers money), it faces the risk of free-riding by individuals who incur little/no cost but gain the benefits of cooperation all the same (Noë, 2010; Olson, 1965). In social groups of any size (from students in a class to large-scale cooperation between countries), cooperation often relies on group norms, laws, policing and punishment to prevent or limit free-riding (Bowles & Gintis, 2011). However, foreseeing the behaviour (cooperation vs. free-riding) of potential social partners, in order to differentiate cooperators from free-riders, is challenging. If cooperators could use quick and reliable phenotypic traits to identify potential social partners who are similarly inclined to cooperate, they would gain the benefit of cooperation without the risk of free-riding (Boyd & Richerson, 1988; Gächter & Fehr, 1999; Olson, 1965). This is particularly true for human cooperation because we often interact with non-familiar individuals, and we live in flexible social environments, whose size and composition vary across time and context (Bowles & Gintis, 2011; Roberts & Sherratt, 1998).

Various hypotheses have been suggested on how phenotypic traits could guide the choice of social partners and cooperation (e.g. Eshel & Cavalli-Sforza, 1982; Gardner & West, 2010; Pepper & Smuts, 2002; Roberts & Sherratt, 2002). The common denominator of these hypotheses is that phenotypic similarity can be used to weigh the cost/benefit of cooperation and to foresee behaviour, that is, phenotypic similarity between two individuals translates into similar behavioural responses (cooperate or free-ride) in those individuals, under conditions requiring cooperation. If phenotypic similarity is a cue for cooperation, cooperators should display phenotypic homophily to minimise the risk of encountering free-riders: they should preferentially join groups composed of, and interact with phenotypically similar individuals to themselves, because these individuals should be more likely to cooperate, other factors being equal (e.g. cost/benefit of cooperation; Eshel & Cavalli-Sforza, 1982; Pepper & Smuts, 2002). Conversely, free-riders should display phenotypic heterophily: they should try to join groups composed of phenotypically different individuals (i.e. cooperators) from themselves to exploit the benefits of cooperation without its associated costs. However, these hypotheses propose different mechanisms that could lead to a positive relationship between phenotypic similarity and cooperation, and focus on different phenotypic traits for promoting cooperation (e.g. Gardner & West, 2010; Roberts & Sherratt, 2002).

One of the most influential hypotheses on phenotypic similarity and cooperation is kin recognition, which allows individuals to preferentially cooperate with their kin and gain inclusive fitness benefits (Hamilton, 1964). Kin recognition often relies on phenotype matching and similarity (Kaminski et al., 2009; Krupp et al., 2012); for example, facial resemblance increases trust in social partners (DeBruine, 2002). Green-beard effects have been proposed to allow phenotypic identification of cooperators among non-relatives, but their importance for human cooperation is debated (Gardner & West, 2010).

Hypotheses based on cultural evolution (e.g. Henrich & McElreath, 2003) provide an alternative mechanism to kin selection and green-beard effects, through which phenotypic similarity (i.e. not necessarily linked to genotypic similarity) can promote cooperation (e.g. Centola et al., 2007; McElreath et al., 2003; McPherson et al., 2001; Ramazi et al., 2016; Tajfel, 1978). Phenotypic traits, like ethnicity, language or religion, can be used as proxies of cultural similarity between two or more individuals, which in turn may indicate their shared adherence to group norms related to cooperation or to punishment of free-riders (McElreath et al., 2003). If so, phenotypic similarity can promote cooperation. For example, phenotypic cues of cultural similarity (e.g. ethnicity) predict the strength and stability of friendship in children (Schneider et al., 1997), adolescents (Joyner & Kao, 2000; Titzmann et al., 2015) and adults (Johnson, 1989). Such a preference for phenotypically similar individuals to ourselves has been observed across cultures and in different age groups (Centola et al., 2007; McPherson et al., 2001).

The phenotypic traits used to identify cultural similarity (e.g. language) often allow for a quick assessment of potential social partners, which is important when there are constraints on how long individuals can familiarise with one another before engaging in cooperative interactions (Centola et al., 2007; McPherson et al., 2001). At the same time, most of these phenotypic traits are inaccurate: speaking the same language or sharing similar religious beliefs does not necessarily predict whether two individuals will cooperate or not. Moreover, a wide range of physical and behavioural traits have been suggested to assess phenotypic similarity, even though some of these traits are experimentally manipulated and have no biological or cultural salience (e.g. Chatman & Flynn, 2001; Dunham et al., 2011; Haslam et al., 1999; Kinzler et al., 2007; Shutts et al., 2010). The extensive literature on minimal group membership indicates that, although group salience has a positive effect on group identity, even simple, non-salient phenotypic traits can trigger ingroup/outgroup biases (e.g. Diehl, 1990; Dunham, 2018; Melamed et al., 2020; Mullen et al., 1992). For example, assigning 5-year-old US children to groups composed of all phenotypically similar members, on the basis of their experimentally assigned t-shirt colour, is sufficient for the emergence of ingroup preference (Dunham et al., 2011). These studies suggest that humans have strong sensitivity for the phenotypic similarity of their group companions, and that they identify themselves more strongly with their group when phenotypic traits can be used to differentiate the ingroup from the outgroup (Mullen et al., 1992). However, it is unclear whether only salient phenotypic traits promote cooperation (e.g. cues that indicate adherence to shared norms related to cooperation and free-riding) or whether phenotypic traits that lack cultural/norm salience can also trigger cooperation (Melamed et al., 2020; Mullen et al., 1992; Sparks et al., 2017).

The importance of the salience of phenotypic traits for cooperation may change during development. Children begin to display an awareness of group membership from around 3 years old, and from around 5 years of age they show an increased preference for individuals or groups with similar phenotypes to themselves (Baron & Banaji, 2006; Kinzler et al., 2007; Kuhlmeier et al., 2014; Nesdale, 2004; Shutts et al., 2010). Similarly to adults, children show ingroup preference in response to non-salient phenotypic traits (Dunham, 2018; Kuhlmeier et al., 2014; Mullen et al., 1992). However, the importance and judgement of phenotypic similarity changes during the course of development (Nesdale, 2004; Rekalidou & Petrogiannis, 2012). For example, unlike adults, 8- to 10-year-old US children consider information about the religious beliefs of an individual to give little information about that individual (Heiphetz et al., 2014). If humans have evolved a broad, non-trait-specific response to phenotypic similarity in relation to cooperation, they should display greater cooperation when in groups composed of all phenotypically similar individuals to themselves than when in phenotypically heterogenous groups. This pattern should also be observed in children, especially in ≥6 year old children, who should have fully developed social categorisation and a response to phenotypic homophily (Nesdale, 2004). Conversely, if While phenotypic homophily is only triggered by specific cues of cooperation, the use of non-salient phenotypic traits would still lead to minimal group membership and ingroup preference in children, including ingroup cooperation (Dunham, 2018; Mullen et al., 1992), but the degree of ingroup similarity should not affect cooperation. In adults, non-salient phenotypic traits might still affect cooperation, because they should have greater experience of using subtle cues of diversity than children (Heiphetz et al., 2014).

In this study, we analysed whether 3- to 10-year-old children and young adults display a preference for, and greater ingroup cooperation towards, members of groups composed of phenotypically similar individuals to themselves. We experimentally manipulated the degree of phenotypic homogeneity in a group in three experiments, using two simple, non-salient phenotypic traits (preferred colour and recreational activity). In the first experiment, we analysed children's preference for phenotypically homogeneous groups (i.e. groups where all members prefer the same colour and recreational activity). We predicted that children would prefer to join a homogeneous group over groups with different degrees of heterogeneity. Moreover, we predicted that children would be more likely to share with members of their chosen group than with other groups (Dunham, 2018; Mullen et al., 1992). In the second and third experiments (respectively on children and young adults), we allocated participants to a phenotypically homogeneous group or to groups with different degrees of heterogeneity (relative to the participant's choice of phenotypic traits; see Methods) and measured their ingroup cooperation. If humans display a broad response to phenotypic similarity/dissimilarity, even when non-salient phenotypic traits are used, participants would be more cooperative in phenotypically homogeneous groups (i.e. groups composed of all participants with similar phenotypic traits to the participant) than in other heterogeneous groups. In children, we tested this hypothesis with the whole cohort of children participants and with ≥6 year old children only, to analyse if phenotypic homophily only emerges in older children (Nesdale, 2004).

## 2. **Methods and** results

### 2.1. Participants

We tested 280, 3 to 10-year-old children (143 girls; mean age ± SE = 7.1 ± 0.12 years; minimum–maximum age = 3.0–10.9 years) for experiment nos. 1 and 2, and 76 young adults (56 women; mean age ± SE = 19.8 ± 0.04 years; minimum–maximum age = 18.1–23.0 years) for experiment no. 3. We collected data on children during the 2017 and 2018 Summer Scientist Week, an event organised each August by the University of Lincoln, where 3 to 10-year old children and their caregivers take part in various studies and recreational activities. We collected data on young adults (i.e. university students) in our computer laboratories. All participants in this study had English as their first language; 95% of participants were white British. This study received ethics approval from the University of Lincoln (reference no. PSY1718266). Data used for this study are available in the University of Lincoln repository [https://eprints.lincoln.ac.uk/id/eprint/55896/].

### 2.2. Experiment no. 1: group preference in children

#### 2.2.1. Data collection – experiment no. 1

**Figure 1.**
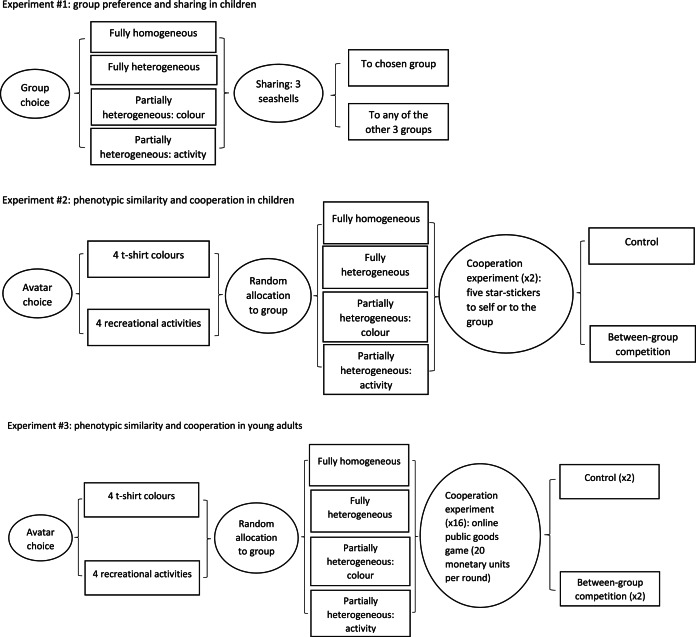
Graphical representation of the sequence (from left to right) of the three experiments. Elliptical shapes represent the key stages of each experiment and rectangular shapes represent the conditions in each stage.

In experiment no. 1, we aimed to analyse whether children preferred to join phenotypically homogeneous groups (i.e. groups where all members prefer the same colour and recreational activity) and were more willing to share a resource with their group companions than with other groups. We asked the children to imagine they were taking part in a sand castle competition between four groups of children; each group was represented by four avatars on a computer screen (see Figure 1 for a graphical description of the key stages and conditions in the three experiments, and Supplementary Material for further details on the experiment and for the scripts and forms given to participants). We told the children that the group that won the competition would get a big prize. The composition of the four groups differed from one another in relation to two phenotypic traits: the avatars’ t-shirt colour (white, yellow, red or blue) and/or their recreational activity (i.e. described preference for a specific TV show: cartoons, movies, animal shows or ‘something else’). Each child was presented with four groups with the following phenotypic composition: (1) one fully heterogeneous group (all avatars in the group with different t-shirt colours and preferences for different TV shows); (2) two partially heterogeneous groups (one group where all avatars had the same t-shirt colour but preferred different TV shows, and one group where all avatars had different t-shirt colours but preferred for the same TV show); and (3) one fully homogeneous group (all avatars in the group with the same t-shirt colour and preference for the same TV show). Having described the composition of the four groups to the children, we asked them to tell us which group they wanted to join. We asked a sub-set of children (*n* = 84) to explain their group choice (Supplementary Material).

After the children had chosen which group to join, we asked them to imagine that they had found three seashells on the beach, that these seashells were very special and everyone wanted them, and that there were no other seashells on the beach. We asked the children some questions to check their comprehension of the experiment (Supplementary Material). Finally, we asked the children to decide how they wanted to distribute the three seashells among the four groups. The researcher entered the number of seashells chosen by the child next to the relevant group, asked the children if they were happy with their choices and submitted their response. At the end of experiment no. 1, we gave the children a 5 minute break, where they could play and relax in the lobby, before starting experiment no. 2 (see below). We ran experiments nos 1 and 2 using the Qualtrics software (Qualtrics, Provo, UT, USA, © 2017).

#### 2.2.2. Data analysis – experiment no. 1

We tested whether the number of children who had chosen one of the four available group compositions as their preferred group was significantly different from a random choice, using a chi-square test. We calculated descriptive statistics for the data on the children's responses (*n* = 84) to the open question about what motivated their group choice.

We used a one sample *t*-test to analyse whether children shared more seashells with their chosen group than what was expected by chance (i.e. 0.75 seashells per group; three seashells in total divided among four groups). Finally, we ran a negative binomial generalised linear model (GLM), using data on 280 children, to test whether the number of seashells children gave to their chosen group (response variable) differed depending on the composition of the chosen group (categorical test fixed effect: fully heterogeneous, fully homogeneous or partially heterogeneous group). We ran a negative binomial GLM because this model had a lower dispersion parameter than a Poisson GLM and a zero-inflated Poisson GLM (see the Supplementary Material). Note that, in this GLM and in the models run with data from the other two experiments, we put together the two partially heterogeneous groups into one single category, so that the group composition variable was composed of three categories. We considered the two phenotypic traits together as analyses run separately on the two traits (colour and recreational activity) gave very similar results (Supplementary Material Tables S5 and S6) to the ones presented below. In this GLM, together with the group composition variable we entered the ages of the children (in years; continuous variable) and their gender (binary variable) as control fixed factors. We ran the analyses in R, version 4.0.3 (R Core Team 2020), with the package ‘glmmTMB’ (Brooks et al., 2017).

For all of the models run for the three experiments, we analysed the collinearity between the fixed factors, with the package ‘car’, using the Variance Inflation Factors (Fox & Weisberg, 2019). The Variance Inflation Factors were always ≤1.10, indicating low collinearity. We compared the full and null model (composed of all control fixed factors in the model except the test fixed effect) for each experiment using a likelihood ratio test. In the results for the three experiments, when the full model was significantly better than the null model, we present the coefficients and *p-*values of the fixed factors in the main text, otherwise the test statistics are presented in the Supplementary Material. Moreover, for each model, we present the results of the control fixed factors, but we do not interpret their effect.

#### 2.2.3. Results – experiment no. 1

**Figure 2.**
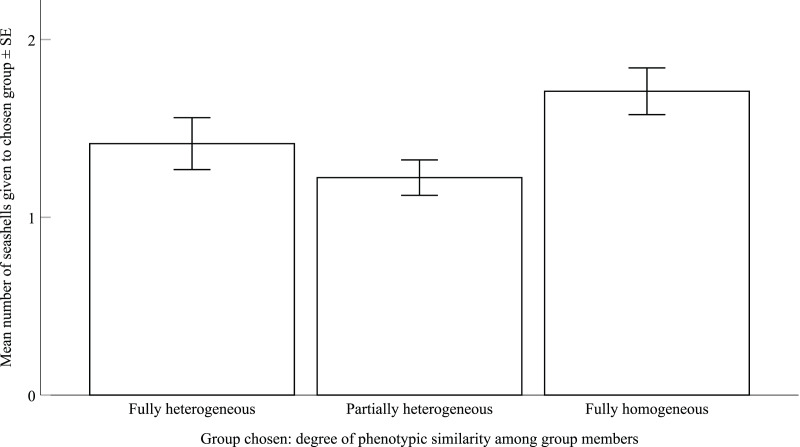
Mean number of seashells (± SE) that children gave to their group companions, divided by the degree of phenotypic similarity of the chosen group.

We found that children's preferences for the three group compositions did not significantly differ from what expected by chance (chi-square test: *χ*^2^ = 1.70, *p* = 0.64; see also Supplementary Table S3). Seventy-nine children (28%) chose the fully homogeneous group, 70 children (25%) chose the fully heterogeneous group and 131 children (47%) chose the partially heterogeneous group. All of the children who chose the fully heterogeneous group focused on the phenotypic diversity of their chosen group (e.g. ‘Boring if everyone is the same’; see full data in Table S1). Among the children who chose the fully homogeneous group, 92% of them focused on the phenotypic similarity of their group. For example, one child said ‘[the members of their chosen group] all like the same things, TV shows and *t*-shirts’. Among the children who chose the partially heterogeneous group, 42% gave a heterophilic and 58% a homophilic response. As predicted, children shared significantly more seashells with their chosen group than what was expected by chance (mean seashells shared by children with their chosen group ± SE = 1.41 ± 0.07; one sample *t*-test, *t* = 9.32, d.f. = 278, *p* < 0.001). Finally, the full negative binomial GLM was not significantly better than the null model, which only excluded group composition (likelihood ratio test: *χ*^2^ = −56.04, d.f. = 2, *p =* 0.98; Figure 2; coefficients and *p*-values for the fixed effects are provided in Supplementary Table S2). To control whether our results were due to the wide age range included in our study, we re-ran the GLM on ≥6-year-old children only. This GLM on the restricted dataset, containing the same factors included in the model above, was not significantly better than the null model (Supplementary Table S4). Therefore, group composition did not predict sharing in children in experiment no. 1.

### 2.3. Experiment no. 2: phenotypic similarity and cooperation in children

#### 2.3.1. Data collection – experiment no. 2

We asked the children to pick an avatar wearing a t-shirt with the colour they liked most, based on their preferred colour, and told them that their chosen avatar would represent them in the game (Supplementary Material). We used the same avatars as in experiment no. 1 and in the same order left to right, in relation to their hair style and colour. Once the children had picked their preferred avatar, we asked them to wear a sports bib with the same colour as their preferred avatar. Moreover, we asked the children to tell us which type of recreational activity (TV show) they liked the most, among the same four options used for experiment no. 1 (i.e. cartoons, movies, animal shows or ‘something else’). We pseudo-randomly allocated children to one of the four groups used in experiment no. 1 (one fully heterogeneous, one fully homogeneous and two partially heterogeneous groups), using the same two phenotypic traits (colour and recreational activity). In this experiment, and in experiment no. 3 on young adults (below), the definition of a group as fully homogenous or partially/fully heterogeneous was always based on which phenotypic traits each child (or young adult) had chosen. For example, when a child was allocated to the fully homogeneous group, the avatars in that group all had the same preference for colour and recreational activity of the avatar chosen by that child.

After some comprehension checks (Supplementary Material), we asked the children to tell us how much they liked their group and how they felt about being in that group, using a five-point smiley face scale. After this, the children played two games, one where there was no competition with another group (control) and one where their group was competing with another fictional group. We used these two conditions to control for whether the predicted greater cooperation in homogeneous groups was affected by outgroup competition (Burton-Chellew et al., 2010; Majolo & Maréchal, 2017; Mullen et al., 1992; Puurtinen & Mappes, 2009). The presentation order of the control and competition conditions was pseudo-randomised across children. In the control condition, we told the children that they had been given five star-stickers that they could exchange, at the end of the experiment, for other stickers of their choice. We asked the children to tell us how many stickers they wanted to keep for themselves and how many (if any) they wanted to give to the other members of their group (i.e. our measure of ingroup cooperation). The competition condition was the same as that of the control, except that we told the children that their group was competing with another group over an extra number of stickers. We did not give the children additional details on the rules of the competition (i.e. the criteria used to determine the outcome of the competition) and on how many stickers they would win/lose, because: (1) the presence of another group with whom to compete (without specific details on the nature of the competition) was sufficient to increase ingroup cooperation in a previous study (Majolo & Maréchal, 2017); and (2) we aimed to keep the experimental rules as simple as possible to avoid comprehension issues with the younger participants. At the end of experiment no. 2, the children picked up some stickers in exchange for their participation (regardless to how many stickers they kept for themselves in experiment no. 2); children and caregivers were debriefed, thanked for their participation and left the laboratory.

#### 2.3.2. Data analysis – experiment no. 2

We ran a generalised linear mixed model (GLMM) with a Poisson error structure and log link function (McCullagh & Nelder, 2019), using the package ‘lme4’ (Bates et al., 2015) in R, version 4.0.3 (R Core Team 2020). The number of stickers that children decided to share with their group companions was the response variable, and group composition (fully heterogeneous, fully homogeneous or partially heterogeneous group) was the categorical test fixed factor. As control fixed factors, we entered the condition (binary: control or competition), ingroup preference, gender and age of the children (in years). Ingroup preference (range = 0–10) was obtained by summing together the scores for the two questions where we asked children how much they liked their group and how they felt about being in that group. Finally, we entered the ID code of the children as a random intercept factor to account for the fact that we had two data points (i.e. competition and control conditions) on each child. The Poisson GLMM was based on data on 280 children and 560 data points.

#### 2.3.3. Results – experiment no. 2

**Figure 3.**
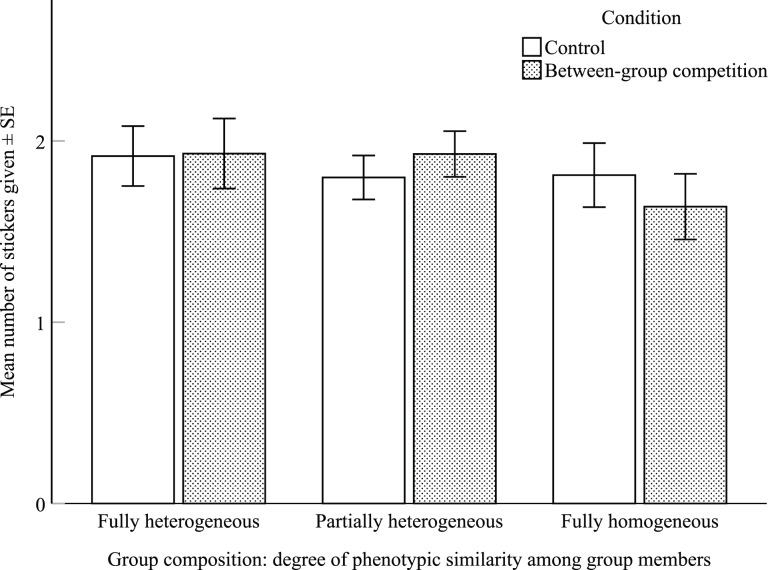
Mean number of stickers (± SE) that children shared with their group companions, in the control and between-group competition conditions, and in the three groups with different degrees of phenotypic similarity between group members.

The full Poisson GLMM, including group composition, condition, ingroup preference, gender and age was not significantly better than the null model, which excluded group composition but was otherwise identical to the full model (*χ*^2^ = 0.46, d.f. = 2, *p =* 0.79; Figure 3; Supplementary Table S5). We obtained the same non-significant difference between the full and null model when we re-ran a Poisson GLMM on ≥6-year-old children only, similarly to what was done for experiment no. 1 (Supplementary Table S). Thus, contrary to our prediction, children did not cooperate more when in a group composed of phenotypically similar members to themselves.

### 2.4. Experiment no. 3: phenotypic similarity and cooperation in young adults

#### 2.4.1. Data collection – experiment no. 3

We recruited young adults among first- and second-year psychology students at the University of Lincoln. We ran an online public goods game (van Dijk & De Dreu, 2021) written using oTree, an open source Python package (Chen et al., 2016), which was hosted as a protected website for the participants to access during the session. Participants read the experiment instructions on the pc screen (Supplementary Material); they also received a hard copy of the instructions that they could check throughout the experiment. In the instructions, we told participants that they were going to play several rounds of an online computer game, involving electronic monetary units (MUs), with other participants who were in the same or in another room. In fact, participants were playing with a set of stooges – generated computer respondents that gave the appearance of other players, including randomised delays in responses in group tasks, as participants had to wait for all ‘players’ to complete a round before moving on. We did not give participants details on how many rounds of the game they had to play to avoid end game effects. To give the MUs a real value, we told participants that they could exchange the MUs that they got during the experiment for a proportional number of credit points that they could use to recruit participants in their final year of their degree. Moreover, the 20 participants with the highest number of MUs entered a prize draw to receive one of four £20 Amazon vouchers. After reading the experiment instructions, participants were given a series of control questions to let them practise the game and to make sure they understood the rules of the experiment (Supplementary Material). We also told participants to ask the researchers in the room if anything was unclear. Once all the participants in the session had completed the control questions correctly and had no questions, we proceeded with the experiment.

At the start of the experiment, we asked the participants their age and gender and asked them to choose the avatar of the colour they liked the most, based on their preferred colour among the ones available (i.e. blue, yellow, red or green); we told the participants that their chosen avatar would represent themselves in the game. Moreover, we asked the participants to tell us which recreational activity they liked the most, among four options (i.e. watching TV, playing sport, playing computer games or hiking). At the start of each round, participants received 20 MUs that they had to allocate, in units of 1, to their private account and/or to the group account (i.e. share with their group companions). The allocation of MUs was anonymous and group members were not informed about how each participant allocated their MUs. Participants could keep MUs in their private account until the end of the experiment. Conversely, the total MUs that group members put in the group account would be multiplied by 2, with a marginal per capita return rate of 0.5 per contributed MU, and then distributed equally among group members, irrespective of their initial contribution. As soon as all group members allocated their MUs, participants were informed about how many MUs they had gained from the group account. For example, if the group put a total of 40 MUs in the group account, each member would get 20 MUs. At the start of each round of the game, we allocated each participant to a group composed of four members; participants could see their allocated group on the computer screen, where participants were represented by avatars, together with a description of the phenotypic traits chosen by the avatars. We used the same group compositions used in experiment no. 2 (one fully heterogeneous, one fully homogeneous and two partially heterogeneous groups). After each round, we changed the composition of the group that each participant was allocated to. As in experiment no. 2, the definition of a group as fully homogenous or partially/fully heterogeneous was based on which phenotypic traits (i.e. colour and activity) each participant had chosen at the start of the experiment.

In half of the rounds, participants were informed that they were competing with another group (competition condition) over an extra amount of MUs: after all group members in the two groups had allocated their MUs, the total numbers of MUs in the group accounts of the two groups were going to be compared. The group with the greater number of MUs in their group account would win the competition. The difference in MUs between the two groups would be doubled: the resulting MUs would be divided equally between members of the winning group, whereas members of the losing group would lose the same amount of MUs. In the control condition, participants were informed that there was another group playing the game with their group, but no additional information was given. The presentation order of the different group compositions and the competition/control conditions was pseudo-replicated across participants. All participants played 16 rounds of the game, so that each of them played the game with all the possible combinations of control/competition condition and group composition: eight rounds with the competition condition and eight rounds with the control condition. In each of these two blocks of eight rounds, participants played two rounds in a fully homogeneous group, two rounds in a fully heterogeneous group and four rounds in a partially heterogeneous group.

After participants allocated their MUs, we asked participants to answer two questions about their allocated group (i.e. how much they wanted to benefit their group and how much they viewed their group companions as collaborators), using a 10-point Likert scale (10 being maximum desire to benefit their group and view the group members as cooperators). This completed the first round. Once participants had completed the 16 rounds of the experiment, they were informed about the number of MUs they had in their private account, as a result of their allocation and that of the other group members. Participants were de-briefed, they exchanged their MUs for a proportional number of credit points and left the laboratory.

#### 2.4.2. Data analysis – experiment no. 3

We ran a negative binomial GLMM, instead of a Poisson GLMM, because this model had lower dispersion (Supplementary Material). For the negative binomial GLMM we used data on 76 young adults and 1216 data points (*N* of participants times *N* of rounds), with the package ‘glmmTMB’ (Brooks et al., 2017) in R, version 4.0.3 (R Core Team 2020). The number of MUs that participants gave to their group was the response variable and group composition (fully heterogeneous, fully homogeneous or partially heterogeneous group) was the categorical test fixed factor. As control fixed factors, we entered condition (binary: control or competition), ingroup preference and gender of the participants. Ingroup preference (range = 0–20) was obtained by summing together the scores for the two questions on how much participants wanted to benefit their group companions and considered them as cooperators. Finally, we added to the negative binomial GLMM random intercepts for participant ID and round, and the random slopes for ingroup preference within participant ID (Supplementary Material for additional details on the choice of random intercepts and slopes).

#### 2.4.3. Results – experiment no. 3

**Figure 4.**
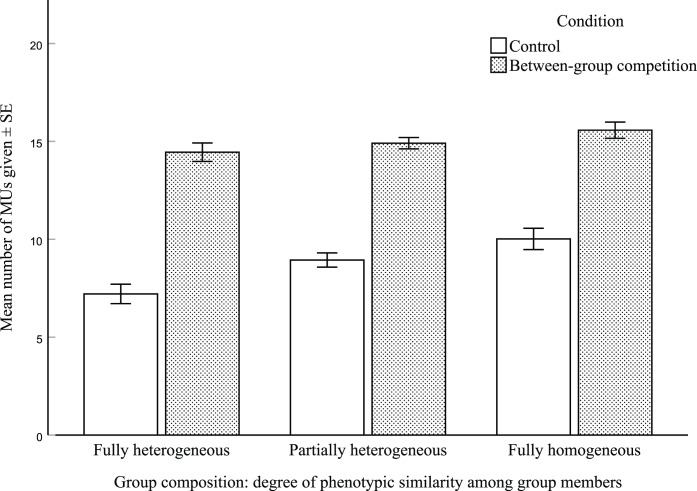
Mean number of monetary units given (± SE) that participants allocated to their group account, in the control and between-group competition conditions, and in the three groups with different degrees of phenotypic similarity between group members.

The full negative binomial GLMM run on young adults (Table 1), comprising group composition, condition, ingroup preference and gender, was significantly better than the null model, which excluded group composition (*χ*^2^ = 17.93, d.f.  = 2, *p* < 0.001). In line with our prediction, ingroup cooperation was affected by the degree of phenotypic similarity in the group (Figure 4). Participants shared on average 12.8 ± 0.4 (SE) MUs with their group companions when they were in a fully homogeneous group, 11.9 ± 0.3 MUs when in a partially heterogeneous group and 10.8 ± 0.4 MUs in a fully heterogeneous group. They also cooperated more in the between-group competition condition (15.0 ± 0.2 MUs shared) than in the control condition (8.8 ± 0.3 MUs). In each round of the game, there was a 17% increased rate of cooperation, on average, when participants were in the fully homogeneous group than in the fully heterogeneous group (rate ratios; Table 1). Moreover, the rate of cooperation increased by 73% when participants when playing the between-group condition instead of the control condition.

**Table 1.** Coefficients, rate-ratio (RR), and *z-* and *p*-values of the fixed factors entered in the negative binomial generalised linear mixed model run with data from experiment no. 3 on young adults. Results for the three pairwise comparisons for the group composition variable were obtained by running two models with different baseline group composition.

Variable	RR	Log-count estimate ± SE	*z*-Value	*p*-Value
Intercept		1.42 ± 0.13	11.32	<0.001
Group composition				
Fully heterogeneous vs. partially heterogeneous	1.09	0.09 ± 0.03	2.72	0.006
Fully homogeneous vs. partially heterogeneous	0.92	−0.08 ± 0.03	−2.44	0.02
Fully heterogeneous vs. fully homogeneous	1.17	0.16 ± 0.04	4.23	<0.001
Condition (control vs. between-group competition)	1.73	0.55 ± 0.03	20.35	<0.001
Ingroup preference	1.07	0.07 ± 0.01	13.58	<0.001
Gender	1.07	0.07 ± 0.14	0.53	0.60

We noticed a potential interaction between group composition and control vs. between-group competition conditions, with group similarity associated with a greater increase in cooperation in the control condition, relative to the between-group competition condition (Figure 4). Therefore, we conducted an exploratory post-hoc analysis to investigate this in more detail. Consistent with our visual interpretation, we found that the rate of cooperation increased by 35% when participants where in the fully heterogeneous group and in the between-group competition condition than when they were in the fully homogeneous group and in the control condition (log-count estimate ± SE = 0.30 ± 0.07, *t* = 4.14, *p* < 0.001; full model results are provided in Supplementary Table S10). We found no significant interaction between group composition and condition for partially heterogeneous vs. fully homogeneous groups (log-count estimate ± SE = 0.07 ± 0.06, *t* = 1.25, *p* = 0.21). The results from this additional model suggest that group similarity may have fostered cooperation more in the control compared with the between-group competition condition (Figure 4). Moreover, it is possible that the main effects reported for the model with no interaction were predominantly driven by the control vs. between-group competition condition.

## 3. Discussion

Our analyses showed that non-salient phenotypic traits can trigger ingroup preference in children, in line with research on minimal group membership (Diehl, 1990; Dunham et al., 2011; Mullen et al., 1992). However, we found no evidence of a preference for, or greater cooperation with phenotypically similar individuals in 3- to 10-year old children. Conversely, in young adults ingroup cooperation increased by an average 13% when participants were in fully homogeneous groups than when in partially or fully heterogeneous groups.

Individual and group categorisation emerge relatively early during development, when children are between 2 and 3 years old, and become more pronounced in older children (Nesdale, 2004). The capacity to differentiate individuals is essential for social partner choice, for the formation of social bonds and of group identity. Such categorisation leads to ingroup preference, whereby individuals prefer to be pro-social and cohesive with members of their own group (e.g. their network of friends; McPherson et al., 2001; Mullen et al., 1992; Nesdale & Lawson, 2011). Ingroup preference is not necessarily linked to outgroup prejudice or hostility (but see below). Supporting this, in our study we found that children were sharing more with their preferred group than with other groups. Such preference for the ingroup may be due to the greater opportunities for direct, indirect and reputation-based reciprocity to be established with group members than with individuals from distinct groups (e.g. Roberts & Sherratt, 1998; Sylwester & Roberts, 2010). At the same time, the categorisation of individuals/groups based on their phenotypic traits should also lead to a preference for phenotypically similar individuals/groups as ourselves (Baron & Banaji, 2006; Kinzler et al., 2007; Shutts et al., 2010), which we do not observe in our study.

Children who were asked to explain why they chose a specific group, in experiment no. 1, often mentioned the importance of phenotypic similarity/dissimilarity of their group. It is possible that these responses where due to demand characteristics and were post-hoc explanations to justify their random group choice. Alternatively, these responses suggest that children in our study population assign different values to group similarity. The type and importance of norms related to inclusivity, conflict avoidance and pro-sociality may change depending on the social context and age of the children (Nesdale & Lawson, 2011; Rizzo et al., 2018), so that children may display a variable preference for phenotypically similar individuals depending on which norm they follow. For example, social inclusivity can both increase and decrease when interactions between groups are common, although intergroup contact typically reduces outgroup derogation (Pettigrew & Tropp, 2006).

As predicted, young adults cooperated more in phenotypically homogeneous groups than in other groups (e.g. Centola et al., 2007; McPherson et al., 2001; Ramazi et al., 2016). A preference for phenotypically similar individuals to ourselves has been observed in different cultures (Johnson, 1989; McPherson et al., 2001). It has been suggested to improve social integration, group cohesion and cooperation, but it can also increase group categorisation, outgroup derogation and conflict (e.g. Haslam et al., 1999; Kinzler et al., 2007; Shutts et al., 2010). Such preference for phenotypically similar individuals, matched with limited opportunities for between-group exchanges, can increase cultural differentiation between groups, with two expected consequences (Bowles, 2009; Zefferman & Mathew, 2015). Firstly, phenotypic homogeneity and reduced between-group contact favour the perception of outgroup individuals as a threat, outgroup de-humanisation and conflict (Bandura et al., 1975; Haslam, 2006; Zefferman & Mathew, 2015). Secondly, when there is between-group conflict, more homogeneous, cohesive and cooperative groups should out-compete more heterogeneous and less cohesive groups (e.g. Bowles, 2009). In our study population, phenotypic similarity may affect cooperation only, or more strongly in the absence of other factors shaping cooperation (such as the presence of a competing group). The relative importance of phenotypic similarity in relation to other drivers of cooperation needs to be investigated further.

Phenotypic homogeneity may be achieved through the use of simple cues, such as age or language (e.g. Haslam et al., 1999; Kinzler et al., 2007; Shutts et al., 2010). These cues can be beneficial in guiding social choices, especially when time constraints or cognitive overload do not allow a more in-depth evaluation of potential social partners (Mellers et al., 1998; Sweller, 1988). In the absence of kin-ties or familiarity between social partners, salient cues of cultural/norm similarity (e.g. language or religious beliefs) should have a stronger effect on cooperation than non-salient cues, because the former should be more reliable predictors of whether individuals cooperate or free-ride (McElreath et al., 2003). Our results on young adults suggest that non-salient phenotypic traits may be sufficient to trigger cooperation. This is in line with previous work indicating that non-salient phenotypic cues affect ingroup preference and group categorisation (Dunham, 2018; Mullen et al., 1992). However, further work is needed to understand what characteristics of a phenotypic trait (e.g. salience, easiness of detection) are most important for social partner choice and cooperation. Most of these traits, including traits that indicate shared adherence to cooperative norms, are not fully accurate and reliable, owing to the risk of encountering norm violators and free-riders. Thus, group members should continuously assess the cooperative attitudes of their social partners to decide whether/with whom to cooperate (Sylwester & Roberts, 2010).

There are several factors that may have affected the different results in children and young adults in our study. Contrary to adults, the children took part in two experiments, always in the same sequence; experiment no. 1 might have primed them and affected how they cooperated in groups of different similarity in experiment no. 2. We ran various comprehension checks with the children, as we did with the young adults (Supplementary Material). However, it is possible that younger children found it harder to follow the experiment. This is an issue that it is hard to reliably ‘solve’ experimentally, because a single experiment used across a wide age group may either be too complex for younger children or too easy for older ones. Despite this, we found similar results in children when we restricted our analyses to older children (≥6 years old), which suggests that our results are unlikely to be biased by a lack of comprehension in younger children. Contrary to the experiment with young adults, the avatars used with children had different hair colours and styles. We added hair colours/styles to the avatars in children to increase the chances that children would react to the avatars as if they were real individuals. However, we cannot rule out the possibility that the avatars’ hair style/colour affected to some extent how children perceived the phenotypic similarities within/across groups, with a possible reduction of the effect of phenotypic traits on cooperation. Finally, it is possible that our experimental design, where groups were fictitious and only shown to participants on a computer screen, may have reduced ecological validity (Winking & Mizer, 2013). However, several studies have used computer-based experiments to analyse ingroup preference and cooperation in children and adults (e.g. Dunham et al., 2011; Sylwester & Roberts, 2010). Thus, it is unlikely that the different response in children and young adults is due to our experimental design.

We still know very little about how the importance of phenotypic traits for cooperation and social partner choice changes during development, so we need to be cautious when explaining the differences between children and young adults found in our study. A previous study (Sparks et al., 2017) showed that 4- to 6-year-old children are more likely to share with photos of recipients who have been described as liking the same activity as the child than a different activity. However, contrary to our study, Sparks and colleagues’ (2017) procedure pointed the children towards the different preferences of the recipients (i.e. they told children that the recipient likes/doesn't like their preferred activity), which may have triggered the observed difference in sharing. Thus, we cannot reliably determine to what extent our findings are due to our experimental procedure or to developmental changes in the importance of non-salient phenotypic traits as cues of similarity and cooperation. Children after the age of 5 years old should have fully developed social categorisation and ingroup preference (Nesdale, 2004). However, our results suggest that children may not perceive non-salient phenotypic traits as relevant for cooperation, contrary to adults, unless the similarities/differences of these traits are clearly pointed to them (Sparks et al., 2017). This difference may be due to the fact that adults have more experience than children at identifying subtle cues of diversity (Heiphetz et al., 2014). Moreover, children up to the age of 10 do not have a fully developed capacity to explain the cause and motivation of other individuals’ behaviour, and may lack the ability to link non-salient phenotypic traits to behavioural responses (cooperation; e.g. Kassin & Pryor, 1985). As discussed above for adults, it is currently unclear how the importance of the characteristics of a phenotypic trait and their effect on cooperation develop in children.

It is important to note that a preference for phenotypically similar individuals can also lead to stable, phenotypically diverse groups when cultural drifts occur (Centola et al., 2007). Clearly, enhancing diversity and inclusivity are imperative goals for our societies, which can reduce prejudice, discrimination and between-group conflict and improve group performance (Levine et al., 2014; Phillips et al., 2009; Sommers, 2006). For example, heterogenous groups of students, in terms of their mathematical abilities, significantly improved the grades of low-ability students in an arithmetic test while not negatively affecting the achievement of the high-ability students (Hooper & Hannafin, 1988). Our results in young adults suggest that it should be possible to use simple, non-salient but more inclusive cues of social identity to promote cooperation, while also reducing outgroup prejudice. This has indeed been tested and demonstrated in the social psychology literature on common ingroup identities (e.g. Gaertner et al., 1993).

In summary, our study showed that phenotypic similarity can increase ingroup cooperation in young adults but not in children. This may be due to adults being exposed to different types of societal norms or being more responsive to subtle cues of diversity, as compared with children. This alludes to the interplay of development and social norms in impacting the presence and relevance of phenotypic similarity on cooperation.

## Data Availability

The data associated with this research are freely available at repository of the University of Lincoln (https://eprints.lincoln.ac.uk/id/eprint/55896/).

## Supplementary material

For supplementary material accompanying this paper visit https://doi.org/10.1017/ehs.2023.25. click here to view supplementary material
