# Supplementary material for: Cooperation and group similarity in children and young adults in the UK
Source: Evol Hum Sci. 2023 Sep 29;5:e29. doi: 10.1017/ehs.2023.25 (PMC10643143; doi:10.1017/ehs.2023.25)
Supplement: Supplementary file 1 [file S2513843X23000257sup001.docx]

**Supplementary material**

**Group similarity and cooperation in children and young adults**

**1. Supplementary methods for experiment #1 and #2 with 3-10 years old children**

For experiments #1 and #2 on children, when caregivers booked a place in the Summer Scientist Week for their child, they received the study brief and consent form to sign, and were asked to provide the child date of birth, gender and tell the organisers whether the child had any special needs. On their arrival at the venue of the Summer Scientist Week, the organisers gave each child a unique ID code that they could use to take part in the studies and activities run at the event. We met the caregivers and children at the entrance of our lab. We recorded the child’s unique ID, which was linked to the data on the child’s age, gender and special needs at the end of the Summer Scientist Week; this procedure allowed us to treat data anonymously. We also briefed again the caregivers and children about the study and gave them the opportunity to ask any question about the study. After this brief, we asked children whether they were happy to take part in the study and, if so, we asked them to seat at a table in the lab, next to the researcher, in front of a desktop computer. We tested one child at a time. The caregivers waited in the lab; they were not in visual contact with their child and were asked not to interfere with the child’s choices during the experiments.

**2. Experiment #1: group preference in children**

2.1 Supplementary methods – Experiment #1

The four groups were displayed all together on the same screen (see section 2.3 below), in an order that was pseudo-randomised across children. The avatars in each group had different hair style and colour; however, across groups the avatars all had the same size on the screen, wore black trousers and had the same range of hair style and colour, and in the same order left to right. The t-shirt colours used in the experiments were white, yellow, red or blue; the TV shows were cartoons, movies, animal shows or ‘something else’. This last, generic category was added in case children did not like any of the other three TV shows options. The colour and TV show used for the fully homogeneous group was pseudo-randomised across children. Our description of the avatars was gender- and age-neutral (see below).

2.2 Text and figures used to describe experiment #1 to children

Comprehension questions:

1. *Do you consider the seashells valuable?* All children answered ‘yes’
2. *Do you think that giving the seashells to a group is a nice thing to do or not?* All children said it was a nice thing to give seashells to a group
3. *Is anything unclear about the game?*

Figure S1: example screenshot of how the four different groups were presented and described to children in experiment #1. The researcher ticked a box (not shown here) next to the group chosen by the children and wrote down the answer, in the box below, about why children chose a specific group. Note that the question “Why did you choose this group?” was only asked to a sub-set of children (n=84; Table S1).


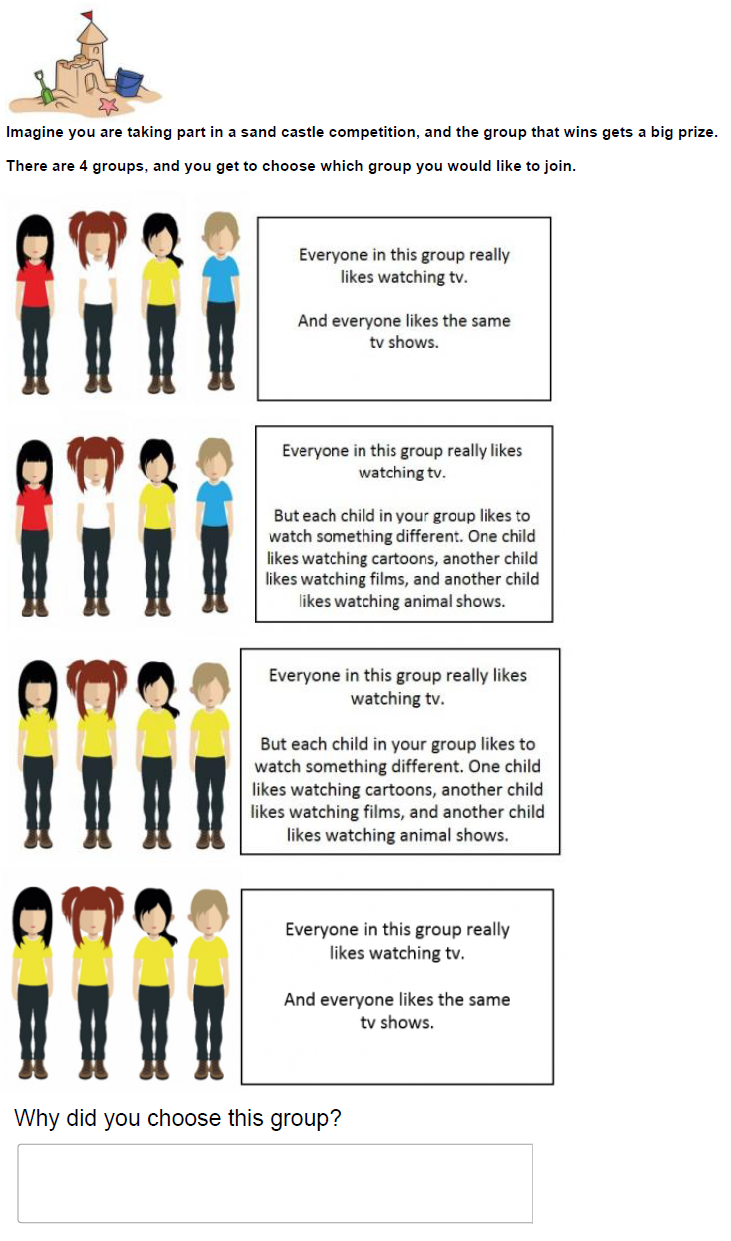


Figure S2: example screenshot of how children were asked about their seashell allocation to the four different group in experiment #1. The researcher entered the number of seashell children gave to each of the four groups in a box (not shown here) next to each group.


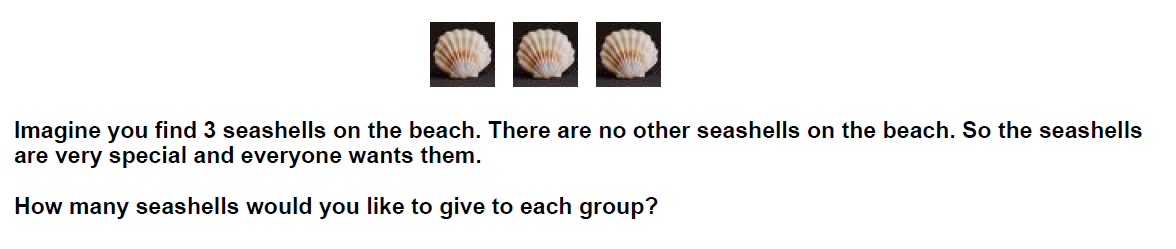


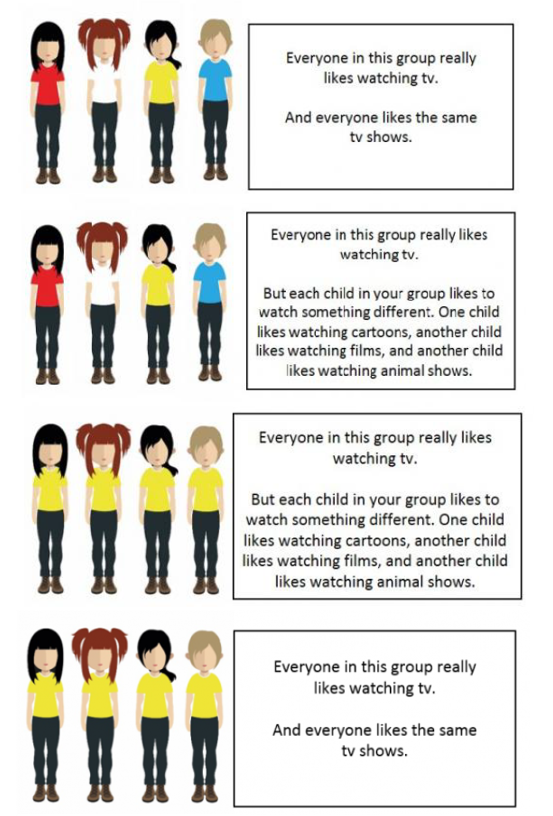


2.3 Supplementary analyses – Experiment #1

We found no significant difference in the number of seashells children game to the two partially heterogeneous group (same colour but different recreational activity vs. different colour but same activity; Wilcoxon test: z=-1.83, p=0.07). Therefore, in the following analyses we combined together these two groups into one partially heterogeneous group.

We first ran a generalized linear model (GLM) with a Poisson error structure and log link function (McCullagh & Nelder, 2019) to test whether the number of seashells children gave to their chosen group differed depending on the composition of the chosen group. Since the Poisson GLM was over-dispersed (dispersion parameter=1.59), we ran a zero-inflated Poisson GLM and a negative binomial GLM with the same variables as the Poisson GLM. The negative binomial GLM had the lowest dispersion parameter (1.14) of the three models and the second best Akaike Information Criterion (AIC) after the Poisson GLM (AIC=70.04 and AIC=67.68, respectively). Thus, in the manuscript we show the results of the negative binomial GLM using data on 280 children. We used the packages ‘MASS’ and ‘pscl’ in R for the GLMs.

To analyse the data on what motivated the children’s group choice (Table S1), two of us independently assigned the responses to one of three categories, whilst being blind about what group the children had actually chosen: heterophylic (when children described their group choice in relation to the phenotypic difference between group members), homophylic (when children described their group choice in relation to the phenotypic similarity between group members) or other (for responses that did not fit within any of the two categories above). Of the 84 children who described to how what motivated their group choice, only 57 responses contained a description that could be analysed. We had almost perfect matching in how the two researchers categorised the responses (Spearman rho = 0.99, p < 0.001): 6 responses were categorised differently by the two researchers and were dropped from the analyses.

Table S1: Responses given by children to the question “Why did you choose this group?” in experiment #1 (see Figure S1 above; FHE = fully-heterogeneous group; FHO = fully-homogeneous group; PHE = partially-heterogeneous group).

| Group chosen | Why did you choose this group? |
| --- | --- |
| FHE | Because I like watching something different |
| FHE | Different colours |
| FHE | Because I like watching animal shows |
| FHE | Everyone likes watching different things |
| FHE | Don't have same colours and everyone is different in tastes which is good for a team |
| FHE | Different colours and like different things |
| FHE | Boring if everyone is the same; different colours and interests |
| FHE | Because everyone likes TV so they can still participate but they all like different shows so can help with answers or whatever is going to be in the competition |
| FHE | Different colours and like different things |
| FHE | Because they are colourful and they have nice hair |
| FHE | I like that they watch different things |
| FHE | Would like to watch different TV shows |
| FHE | Because I like the colours and I like the choices of TV |
| FHE | All are different and not always the same thing. Because everyone is different to you and you are different to every other person |
| FHE | Because normally when I watch TV, I normally watch things like the chase or EastEnders |
| FHE | Because they watch different TV shows |
| FHE | They like watching different shows |
| FHE | Because I like watching TV too and I like a variety of different shows so it will be a good idea to have lots of people who like different shows and they all have different tops |
| FHE | Because everyone watches different things |
| FHE | Original team |
| FHE | Different colours |
| FHE | Wearing individual colours and they like watching different things |
| FHE | Watch different TV shows |
| FHE | Likes rainbow colours and they all like different things |
| FHE | Because original team |
| FHE | Different coloured t-shirts |
| FHE | Really like watching TV |
| FHO | Because I like yellow |
| FHO | I really like yellow |
| FHO | Because they watch all the same TV shows |
| FHO | Because I would rather have everyone liking the same show rather than choosing one as it could cause an argument |
| FHO | Like watching same things |
| FHO | All like watching same thing |
| FHO | Because that is the group I was in and I don't want anyone to be the odd one out |
| FHO | All wearing blue and like same things |
| FHO | Because it has got all yellow tops |
| FHO | If you have a friend who watches the same TV shows then you can talk about them together |
| FHO | Because it's white team and it's the same as England t-shirts |
| FHO | I want to be that one |
| FHO | Wear same t-shirts so they like each other and they like watching the same thing |
| FHO | They all like the same things, TV shows and t-shirts |
| FHO | Because they watch the same things |
| FHO | Because they don't watch the same shows |
| FHO | I like watching same things |
| PHE | Because I might not like the same shows |
| PHE | Because if you'd want to watch a show on the TV you can all watch the same one, because it’s your favourite; I also don't like all yellow |
| PHE | Because all of them like watching different shows, but if there was one without animal shows I'd choose that one because I don't like animal shows |
| PHE | Because it wouldn't be fair if we couldn't all watch the same thing at the same time, because people would complain at the other people |
| PHE | Because there can't be any arguing about what you watch as you all like the same thing |
| PHE | Because they are different |
| PHE | Because we all like to watch the same and agree on stuff |
| PHE | Have different colours |
| PHE | Best group they work together |
| PHE | Because that's my favourite; I like red: red is like pink |
| PHE | All like the same TV shows so they will get along. If they like different things then they might fight. Coloured t-shirts might represent their personalities |
| PHE | Like TV |
| PHE | Because they are not the same colour and they love the same TV shows |
| PHE | Because its fun, the white team is fun |
| PHE | Same colour as my own t-shirt |
| PHE | Because you like what you like and you might want to be with someone who likes you so you don't feel different to them |
| PHE | I like to watch something different |
| PHE | Would like everyone to watch the same TV shows |
| PHE | All like the same shows |
| PHE | Everyone likes watching something different |
| PHE | Same t-shirt colour as me |
| PHE | Like the coloured t-shirts |
| PHE | Because I just watch the same thing all the time |
| PHE | Everyone likes the same TV shows |
| PHE | Random choice |
| PHE | Because they have different t-shirts |
| PHE | Because they are red |
| PHE | Because it shows that you are different and I think I would like to do it. Because it shows that everyone is an individual instead of being all the same |
| PHE | Because me and my sister like watching the same programme |
| PHE | I like watching animal shows; it would help for competition |
| PHE | Because I like watching things different |
| PHE | Because they're all wearing blue |
| PHE | Really like it |
| PHE | Because that is my team |
| PHE | Everyone watching different shows so they will be able to build the sandcastle better, because they have more knowledge as a team |
| PHE | Because they watch animal shows |
| PHE | Because I like watching the same TV shows |
| PHE | Because they are all red and I like watching different stuff to other people |
| PHE | Because if you like watching all the same shows you can all be friends; if they all came to your house and wanted to watch TV they can all decide what to watch so there wasn't a big argument |
| PHE | Don't want to be the same, I want to be unique |

2.4 Supplementary results – Experiment #1

Tests statistics for the full model (see main manuscript) and for the model run with data on ≥ 6 old children only. Both these models were not significantly better than their null model.

R script for the negative binomial generalized linear model on children, presented in Table S2:

full.nb=glm.nb(sea.groupchosen ~ group3.exp1 + gender + age,

data=test.data)

Table S2: Coefficients, z and p values of the fixed factors entered in the negative binomial generalized linear model run with data from experiment #1 on children.

| Variable | Estimate ± SE | z value | p value |
| --- | --- | --- | --- |
| Intercept | -0.57 ± 1.08 | -0.53 | 0.59 |
| Group composition |  |  |  |
| Fully heterogeneous vs. partially heterogeneous | 0.33 ± 0.55 | 0.06 | 0.55 |
| Fully heterogeneous vs. fully homogeneous | -0.07 ± 0.56 | -0.13 | 0.90 |
| Gender | 0.31 ± 0.47 | 0.68 | 0.49 |
| Age | -0.01 ± 0.12 | -0.02 | 0.98 |

Table S3: Coefficients, z and p values of the fixed factors entered in the negative binomial generalized linear model run with data from experiment #1 on children, where group composition is composed of four categories (i.e. fully homogeneous, partially heterogeneous for colour, partially heterogeneous for activity and fully heterogeneous).

| Variable | Estimate ± SE | z value | p value |
| --- | --- | --- | --- |
| Intercept | -0.59 ± 1.51 | -0.40 | 0.70 |
| Group composition |  |  |  |
| Fully heterogeneous vs. partially heterogeneous - colour | 0.38 ± 0.88 | 0.43 | 0.67 |
| Fully heterogeneous vs. partially homogeneous - activity | 0.27 ± 1.05 | 0.26 | 0.80 |
| Partially heterogeneous – colour vs. partially heterogeneous - activity | -0.10 ± 1.25 | -0.08 | 0.94 |
| Fully homogeneous vs. partially heterogeneous - colour | -0.45 ± 0.95 | -0.48 | 0.63 |
| Fully homogeneous vs. partially homogeneous - activity | -0.35 ± 1.15 | -0.31 | 0.76 |
| Fully heterogeneous vs. fully homogeneous | -0.08 ± 0.66 | -0.12 | 0.90 |
| Gender | 0.28 ± 0.62 | 0.47 | 0.64 |
| Age | 0.01 ± 0.15 | 0.03 | 0.97 |

Table S4: Coefficients, z and p values of the fixed factors entered in the negative binomial generalized linear model run with data from experiment #1 on ≥ 6 old children only.

| Variable | Estimate ± SE | z value | p value |
| --- | --- | --- | --- |
| Intercept | 0.48 ± 2.73 | 0.18 | 0.86 |
| Group composition |  |  |  |
| Fully heterogeneous vs. partially heterogeneous | 0.95 ± 0.81 | 1.15 | 0.25 |
| Fully heterogeneous vs. fully homogeneous | 0.41 ± 0.75 | 0.57 | 0.57 |
| Gender | -0.45 ± 0.72 | -0.62 | 0.53 |
| Age | -0.02 ± 0.26 | -0.06 | 0.96 |

**3. Experiment #2: phenotypic similarity and cooperation in children**

Comprehension questions:

1. *Do you consider the stickers valuable?* All children answered ‘yes’
2. *Do you think that giving the stickers to a group is a nice thing to do or not?* All children said it was a nice thing to give seashells to a group
3. *Is anything unclear about the game? Do you have any question?*

Figure S3: screenshot of the practice questions given to children at the start of experiment #2.


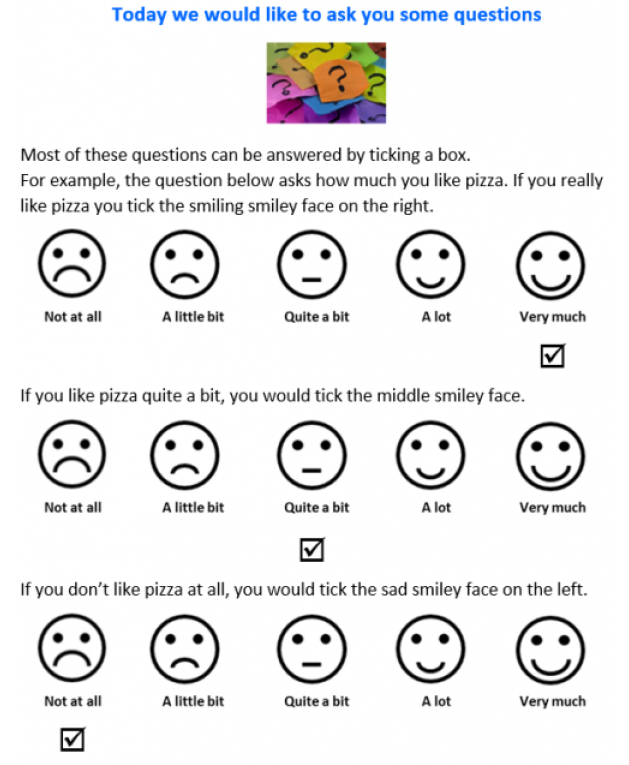


Figure S4: example screenshot of how the children were introduced to their group (in this example, a fully homogeneous group; see main manuscript) in experiment #2, and how they were asked about their ingroup preference. The researcher ticked the circle below the face chosen by the children.


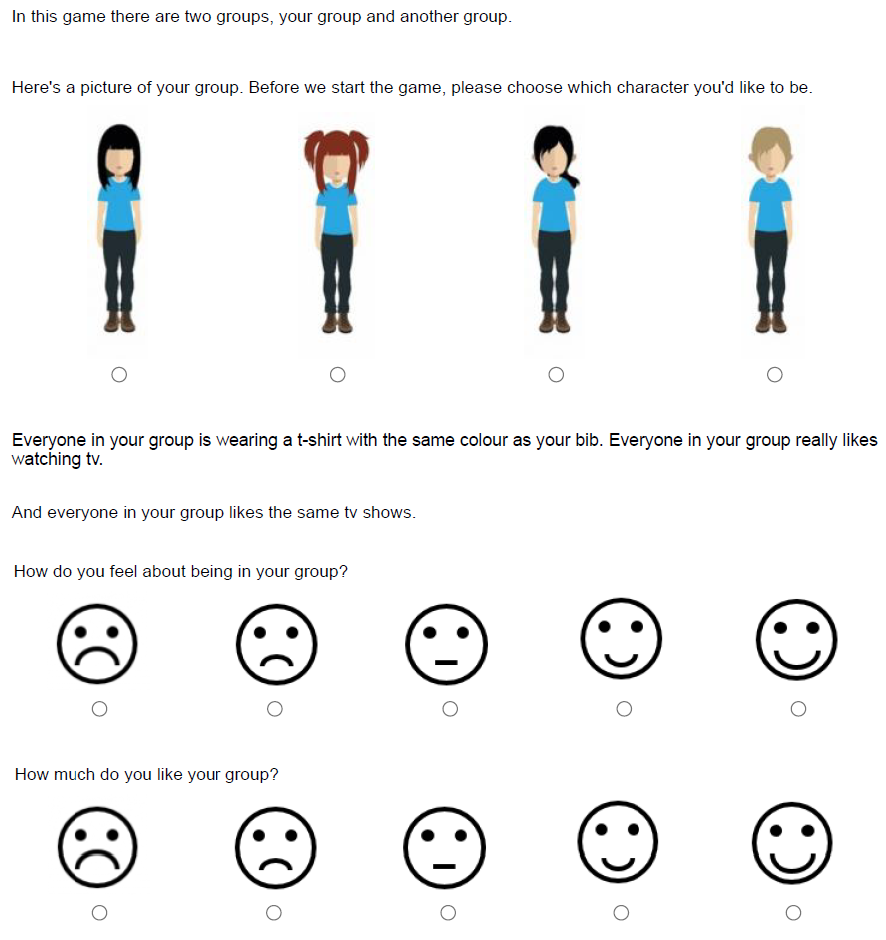


Figure S5: example screenshot of how children were asked about their allocation of star stickers in experiment #2 (in the example, a fully homogeneous group in the control condition). The researcher entered the number of stickers in the boxes on the right, up to a total of five stickers.


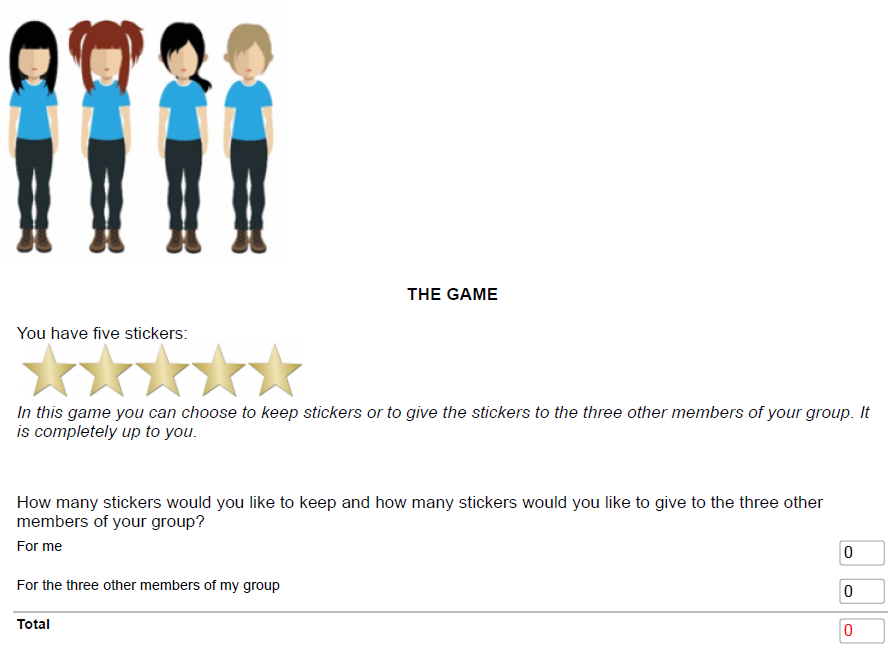


Figure S6: example screenshot of how children were asked about allocation of star stickers in experiment #2 (in the example, a fully homogeneous group in the between-group competition condition). The researcher entered the number of stickers in the boxes on the right, up to a total of five stickers.


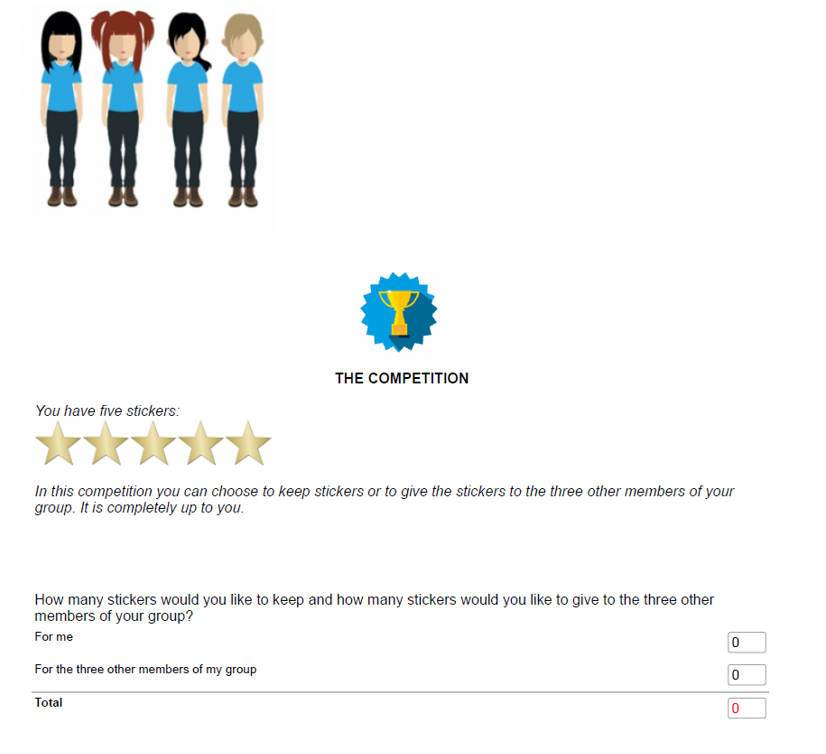


3.1 Supplementary results – Experiment #2

Tests statistics for the full model (see main manuscript) and for the model run with data on ≥ 6 old children only. These models were not significantly better than their null model.

R script for the Poisson generalized linear mixed model on children, presented in Table S4:

full=glmer(stickersgroup.exp1 ~ groupcomp.ac + condition.exp2

+ ingroupref + gender + age +

(1|id.code),

family=poisson, data=xdata)

Table S5: Coefficients, z and p values of the fixed factors entered in the Poisson generalized linear mixed model run with data from experiment #2 on children.

| Variable | Estimate SE | z value | p value |
| --- | --- | --- | --- |
| Intercept | -0.39 ± 0.35 | -1.12 | 0.26 |
| Group composition |  |  |  |
| Fully heterogeneous vs. partially heterogeneous | -0.05 ± 0.11 | -0.44 | 0.66 |
| Fully heterogeneous vs. fully homogeneous | -0.08 ± 0.13 | -0.64 | 0.52 |
| Condition (control vs. between-group competition) | 0.01 ± 0.06 | 0.22 | 0.83 |
| Ingroup preference | -0.04 ± 0.03 | -1.29 | 0.20 |
| Gender | 0.13 ± 0.09 | 1.43 | 0.15 |
| Age | 0.16 ± 0.02 | 6.68 | < 0.01 |

Table S6: Coefficients, z and p values of the fixed factors entered in the Poisson generalized linear mixed model run with data from experiment #2 on children, where we test if the colour trait affects cooperation. In this model the group composition condition is a binary variable: same colour vs. different colour.

| Variable | Estimate SE | z value | p value |
| --- | --- | --- | --- |
| Intercept | -0.64 ± 0.39 | -1.66 | 0.10 |
| Group composition (same vs. different colour) | 0.04 ± 0.09 | 0.50 | 0.62 |
| Condition (control vs. between-group competition) | 0.01 ± 0.06 | 0.22 | 0.83 |
| Ingroup preference | -0.04 ± 0.03 | -1.29 | 0.20 |
| Gender | 0.13 ± 0.09 | 1.40 | 0.16 |
| Age | 0.16 ± 0.02 | 6.698 | < 0.001 |

Table S7: Coefficients, z and p values of the fixed factors entered in the Poisson generalized linear mixed model run with data from experiment #2 on children, where we test if the recreational activity trait affects cooperation. In this model the group composition condition is a binary variable: same recreational activity vs. different recreational activity.

| Variable | Estimate SE | z value | p value |
| --- | --- | --- | --- |
| Intercept | -0.39 ± 0.39 | -1.02 | 0.31 |
| Group composition (same vs. different recreational activity) | -0.13 ± 0.09 | -1.42 | 0.16 |
| Condition (control vs. between-group competition) | 0.01 ± 0.06 | 0.22 | 0.83 |
| Ingroup preference | -0.04 ± 0.03 | -1.25 | 0.21 |
| Gender | 0.13 ± 0.09 | 1.43 | 0.16 |
| Age | 0.16 ± 0.02 | 6.64 | < 0.001 |

Table S8: Coefficients, z and p values of the fixed factors entered in the Poisson generalized linear mixed model run with data from experiment #2 on ≥ 6 old children only.

| Variable | Estimate SE | z value | p value |
| --- | --- | --- | --- |
| Intercept | -0.13 ± 0.38 | -0.34 | 0.73 |
| Group composition |  |  |  |
| Fully heterogeneous vs. partially heterogeneous | -0.09 ± 0.10 | -0.94 | 0.35 |
| Fully heterogeneous vs. fully homogeneous | -0.07 ± 0.12 | -0.57 | 0.57 |
| Condition (control vs. between-group competition) | 0.02 ± 0.07 | 0.31 | 0.76 |
| Ingroup preference | -0.01 ± 0.03 | -0.24 | 0.82 |
| Gender | 0.05 ± 0.08 | 0.58 | 0.56 |
| Age | 0.10 ± 0.03 | 3.36 | < 0.001 |

**4. Experiment #3: phenotypic similarity and cooperation in young adults**

4.1 Supplementary methods – Experiment #3

We collected data over three days in January 2019, across six sessions (two sessions per day). In each session we had an average of 12.2 participants (range 9-19) taking part in the experiment at the same time in a large computer room. Thirty minutes before a scheduled session we greeted the participants in the lobby and gave them the consent form. Participants who consented to take part in the study were allocated to specific desks in the computer room; participants were separated by at least 2m from one another and their desk had separation screens, so that participants could not see the computer screen of the other participants in the room.

4.2 Instructions to participants – Experiment #3

These instructions were shown to participants on a pc screen, but we also gave participants a hard-copy of the instructions, so they could check these, if necessary, throughout the experiment.

Dear participant,

Thank you very much for your participation in this study!

This study should take around 30 minutes. First, we will ask you some questions about you. We will then give you the instructions for the game you are about to play. If the instructions are not clear, please raise your hand and the experimenter in the room will come to address any question you may have. It is very important that you fully understand the rules of this game. It is also very important that you take this experiment seriously and do not get distracted whilst playing the game.

Game instructions:

1. There are several participants to this experiment, in this room or in a room on another floor. All the participants are connected via their computers; you will play this game with some of the other participants.
2. You will play several rounds of this game. Your decisions in each round, and your answers to the questions, will be anonymous.
3. For the whole duration of this experiment, you will be represented by an avatar of your chosen colour and recreational activity; the same will happen to the avatar of the other participants, depending on their choice of colour and recreational activity.
4. At the start of each round of the game, you and the other participants will be randomly allocated to groups of four participants. Once the groups have been composed, you will see the composition of your group, represented by the avatars of the three other members of your group and of yourself. The same information on group composition will be available to the other members of your group and to the other participants.
5. The composition of your group and of the other groups will change after each round.
6. At the start of each round, you will receive 20 electronic monetary units (MUs). You have two different accounts: your private account and a group account. You will need to decide how many MUs (if any) you want to put in your private account and how many MUs (if any) you want to put in the group account.
7. Your allocation of MUs must be in units of 1, that is, you can put 3 MUs in one account, for example, but not 3.1 MUs. You must use all the 20 MUs on each round so that your MUs must be put in your private account and/or in the group account. Remember: your decision is anonymous and for us it makes no difference whether/how many MUs you put in each of your two accounts.
8. Any MUs you decide to put in your private account (if any) are yours and nobody can take them from you. For example, if you put 1 MUs in your private account, this 1 MU will stay in your account until the end of the experiment.
9. Any MUs you decide to put in the group account (if any) will give a return in the following manner: the contributions of all will be summed together using a rate of 0.5 MUs per 1 MU contributed and then distributed equally to all players in the group.
10. Once all the members of your group have allocated their MUs to the two accounts (their private account and the group account), you will be able to see how many MUs you have received from the group account.
11. Together with the change in group composition, there is another condition that can change across rounds, that is, whether your group is competing with another group or not.
12. In the no-group competition condition, there is no additional information you need, and you can start playing the game.
13. Conversely, in the between-group competition condition you and the other members of your group will be informed at the start of the round that you have been randomly selected to compete with another group, randomly selected among the other groups. You will play the game according to the rules described above. However, at the end of the round the total number of MUs in the group account of your group, and of the competing group, is going to be compared. The difference in MUs between the two groups is going to be doubled and divided equally between members of the winning group (i.e. the group with the greater number of MUs in their group account). The losing group (i.e. the group with the smaller number of MUs in their group account) will lose the same amount of MUs. For example, imagine that group W has put a total of 4 MUs in their group account and group X has put 2 MUs in their group account. If so, group W has won the competition. This means that each member of group W will receive 1 MU (4 – 2 = 2 MUs; 2 * 2 = 4 MUs divided equally among the four group members) in their private account whereas each member of group B will have a deduction of 1 MUs in their private account.
14. To sum up, at the start of each round we will inform you about the composition of your group and about whether your group is competing with another group or not. Thus, please carefully read the instructions of the game at the start of each round.
15. Once you have been informed about the outcome of the round, the MUs you got from the group account and from your private account will be put in a separate account until the end of the game.
16. You will be asked to answer two questions about your view of your group. Please remember that your decisions and answers are anonymous.
17. These questions complete the first round of the game. You will then play another round of the game and you will be informed, at the start of the new round, about the composition of your group and about whether your group plays the between-group competition condition or the no-competition condition.
18. You will play an indefinite number of rounds of this game.
19. Once you complete the experiment, you will be de-briefed and can leave the lab.
20. We will give you 2 credit points as a thank you for your participation.
21. Moreover, at the end of the game we will calculate the total number of MU you have earned (MUs that you have put in your private account + MU that you have received from the group account). This sum will be exchanged for credit points according to these rules: participants who have 0-49 MUs will get no extra credit points, participants who have 50-149 MUs receive 1 credit point, participants who have 150-249 MUs receive 2 credit points and so on.
22. Finally, the 20 participants who got the top 20 highest number of MUs at the end of the game (sum of MUs in their private account + earnings from the group account), at the end of the experiment, will be entered in a prize draw to receive one of four £20 Amazon vouchers.
23. If everything is clear, please press the button so you can start the experiment, otherwise please raise your hand and one of the researchers will come to help you. At the start of the experiment we will ask you a few questions to make sure the rules of the games are clear. You will also have a chance to play some practice rounds of this game, before starting the actual experiment. If you are still unsure about the game then, please ask one of the researchers in the room to provide additional details or clarifications on any unclear aspect of the experiment.
24. We hope you enjoy this experiment; thank you very much for your participation!

4.3 Practice questions given to participants at the start of experiment #3

Let’s have some practice on the rules of this game to make sure everything is clear!

Imagine there is a group composed of four members: A, B, C and D. Each player has 20 MUs. In the table below you can see how they allocated their MUs to their private account and to the group account.

Remember: any MUs each player decides to put in their private account (if any) is theirs and nobody can take that away from them.

Also, please remember: any MUs each player decides to put in the group account (if any) will give a return of 0.5 MUs to every member of your group, regardless to whether they have put some/all their MUs to the group account or not.

So, please try to calculate how many MUs each player will have in their private account at the end of this game, by entering their outcome in the boxes on the right:

| Player | Private account | Group account | Total number of MUs in the group account | Reward of MUs from the group account for each player | Outcome (MUs in private account + from group account) |
| --- | --- | --- | --- | --- | --- |
| A | 10 | 10 |  |  |  |
| B | 0 | 20 |  |  |  |
| C | 20 | 0 |  |  |  |
| D | 10 | 10 |  |  |  |

Here are the correct answers:

| Player | Private account | Group account | Total number of MUs in the group account | Reward of MUs from the group account for each player | Outcome (MUs in private account + from group account) |
| --- | --- | --- | --- | --- | --- |
| A | 10 | 10 | 40 | 20 (i.e. 40 * 0.5) | 30 (i.e. 10+20) |
| B | 0 | 20 |  | 20 (i.e. 40 * 0.5) | 20 (i.e. 0+20) |
| C | 20 | 0 |  | 20 (i.e. 40 * 0.5) | 40 (i.e. 20+20) |
| D | 10 | 10 |  | 20 (i.e. 40 * 0.5) | 30 (i.e. 10+20) |

What that easy to calculate and clear? If not, please have another go or ask the researcher in the room to go through the scores with you.

Now, imagine the group above (let’s call it W) is playing this round under the between-group competition condition with another group (let’s call it X).

Remember: in the between-group competition condition, two groups will be randomly paired. At the end of the round the total number of MUs in the group account of the two groups is compared. The group that ends up having the greater number of MUs in the group account, between the two competing groups, would earn double the difference between the contribution to the common group project of their group and that of the other group, whereas the loser group will have a loss of the same amount.

Members of group W have put 15 MUs in the group account (see table above). Now imagine that members of group X have put 11 MUs in their group account. Group W has won the competition and group X has lost it.

Please write in the boxes below how many MUs (if any) each member of the two groups receives or loses based on the outcome of the between-group competition:

Here are the correct answers:

Each member of group W has a reward of 2 MUs and a loss of 0 MUs.

Each member of group X has a reward of 0 MUs and a loss of 2 MUs.

This is how the wins/losses are calculated: W has won the competition, so each member gets an equal share of double the difference of their group’s contribution to their group account and that of the other group (i.e. 15 – 11 = 4; 4 * 2= 8 MUs divided among the four group members). Group X has lost the competition, so each group member has a loss of 2 MUs.

What that easy to calculate and clear? If not, please have another go or ask the researcher in the room to go through the scores with you.

4.4 Initial questions asked to each participant at the start of the experiment, after they received the instructions and did some practice sessions (above).

1. What is your age (in years)?
2. You identify your gender as (tick as appropriate):

Female, Male, Trans, Gender Variant/Non-conforming, Not listed, Prefer not to say

1. What is your favourite colour among the following options (tick as appropriate)?

Blue, Red, Green, White, Black

1. What is your favourite recreational activity among the following options (tick as appropriate)?

Watching TV, Playing sport, Playing computer games, Hiking

4.5 Question about resource allocation decision. In the experiment, the “###” was replaced, by the oTree package, by the relevant colour (i.e. Blue, Red, Green, White or Black) and recreational activity (i.e. Watching TV, Playing sport, Playing computer games or Hiking) depending on the group composition (i.e. fully homogeneous, partly heterogeneous or fully heterogeneous) of each round.

You have chosen ### as your preferred colour and ### as your preferred recreational activity. The other three members of your group have made the following choices for their preferred colour and recreational activity: ###. You can see the avatar of the other three group members and of yourself on the top-left side of the screen.

In this round, you are playing the (shown as appropriate): no-competition condition / the between-group competition.

Please remember that the experiment instructions are available on the document you have been given at the start of the experiment; please consult the instructions any time you want during the experiment.

You have 20 MUs, and you need to decide how many (if any) MUs you want to put in your private account and how many MUs (if any) you want to put in the group account.

Please write down the number of MUs you want to put in one/both of your accounts in the relevant boxes below.

4.6 Questions about the participants’ ingroup preference, given to participants at the end of each round, after the resource allocation question (above).

1. To what extent your decision on how many MUs to put in your private account and in the group account was aimed at benefitting your group?

10-point scale: 0=not based on this; 10= really based on this

1. After this round, how do you consider the other members of your group?

10-point scale: 0=collaborator; 10=competitor

Figure S7: example of the avatars used to describe the group composition in experiment #3 for the different colours (Blue, Red, Green, White or Black).


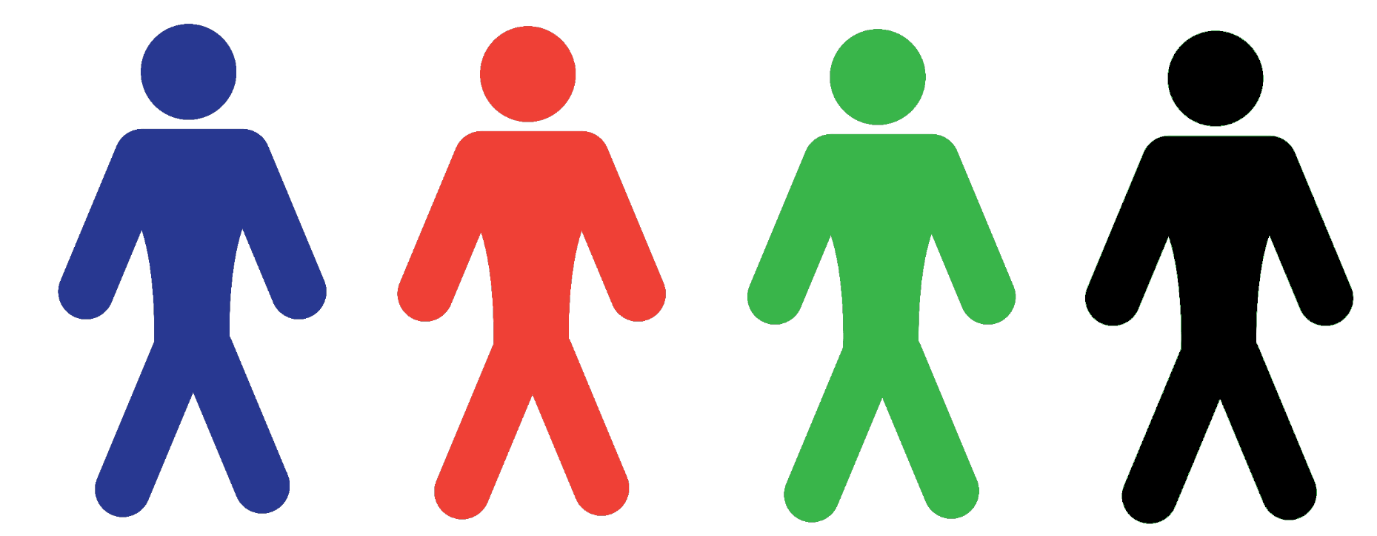


4.7 Supplementary analyses – Experiment #3

We first ran a Poisson GLMM where the number of tokens participants gave to their group was the response variable and group composition (fully heterogeneous, fully homogeneous or partially heterogeneous group) was the categorical test fixed factor. As control fixed factors, we entered condition (binary: control or competition), ingroup preference and gender of the participants. Ingroup preference (range=0-20) was obtained by summing together the scores for the two questions on how much participants wanted to benefit their group companions and considered them as cooperators. Initially, we entered participant ID and round of the game as two random intercept factors, the random slopes of group composition and ingroup preference within participant ID and round, and the correlation between slopes and intercepts (Barr et al., 2013). However, the model did not reach convergence. So, we simplified the model by removing estimates of the terms that were close to 1 and thus not identifiable (Matuschek et al., 2017). The resulting model, which reached convergence, included the random intercepts for participant ID and round, and the random slopes for ingroup preference within participant ID. Since the Poisson GLMM was over-dispersed (dispersion parameter=1.49) but not zero-inflated, we ran a negative binomial GLMM, which fixed the over-dispersion issue (dispersion parameter=1.02). The model fit of the negative binomial GLMM was better than the fit of the Poisson GLMM (AIC = 7246.5 and AIC = 7478.8, respectively). Thus, in the manuscript we present the results of the negative binomial GLMM.

4.8 Supplementary results - Experiment #3

R script for the negative binomial generalized linear mixed model on young adults, presented in Table 2:

full.nb=glmmTMB(contribution ~ groupcomp.ac + condition + ingrouppref + gender

+(1|participant)+(0+ingrouppref|participant)

+ (1|round),

family=nbinom2 (link="log"), data=xdata)

Table S9: Coefficients, z and p values of the fixed factors entered in the negative binomial generalized linear mixed model run with data from experiment #3 on young adults, where we test if the colour trait affects cooperation. In this model the group composition condition is a binary variable: same colour vs. different colour.

| Variable | Estimate ± SE | z value | p value |
| --- | --- | --- | --- |
| Intercept | 0.66 ± 0.22 | 3.02 | 0.003 |
| Group composition | 0.01 ± 0.03 | 0.22 | 0.83 |
| Condition (control vs. between-group competition) | 0.53 ± 0.03 | 19.33 | <0.001 |
| Ingroup preference | 0.07 ± 0.01 | 13.50 | <0.001 |
| Gender | 0.11 ± 0.11 | 0.93 | 0.35 |

Table S10: Coefficients, z and p values of the fixed factors entered in the negative binomial generalized linear mixed model run with data from experiment #3 on young adults, where we test if the colour trait affects cooperation. In this model the group composition condition is a binary variable: same recreational activity vs. different recreational activity.

| Variable | Estimate ± SE | z value | p value |
| --- | --- | --- | --- |
| Intercept | 0.69 ± 0.22 | 3.12 | 0.002 |
| Group composition (same vs. different recreational activity) | -0.01 ± 0.03 | -0.36 | 0.72 |
| Condition (control vs. between-group competition) | 0.53 ± 0.03 | 19.33 | <0.001 |
| Ingroup preference | 0.07 ± 0.01 | 13.50 | <0.001 |
| Gender | 0.11 ± 0.11 | 0.93 | 0.35 |
